# Occipito-cervical fusion following gross total resection for the treatment of spinal extramedullary tumors in craniocervical junction: a retrospective case series

**DOI:** 10.1186/s12957-015-0689-0

**Published:** 2015-09-18

**Authors:** Hua Jiang, Juliang He, Xinli Zhan, Maolin He, Shaohui Zong, Zengming Xiao

**Affiliations:** Department of Spine Surgery, The First Affiliated Hospital of Guangxi Medical University, Shuangyong Road No.6, Nanning, 530021 China

**Keywords:** Craniocervical junction, Extramedullary tumor, Intraspinal tumor, Occipito-cervical fusion

## Abstract

**Background:**

Previous studies found that the facet joint of the C1 vertebra were removed (C1 facetectomy) before extirpation from the extramedullary tumor in craniocervical junction, leading to postoperative upper cervical instability or deformity. Occipito-cervical fusion (OCF) is a demanding and morbid surgical procedure, which can be used in such patients. This study is to analyze the clinical manifestation and surgical outcome of patients with craniocervical extramedullary tumor undergoing an extirpation of spinal tumors and OCF by one-stage posterior approach.

**Methods:**

The surgical and clinical databases were searched for operative procedures that had been performed for patients with spinal extramedullary tumors in craniocervical junction at a single institution from January 2008 to July 2011. The following inclusion criteria were applied: (1) initial surgery for craniocervical extramedullary tumor, (2) gross total resection and occipito-cervical fusion had been performed, (3) minimum 2-year follow-up, and (4) no previous cervical spine surgery. Medical records included demographic characteristics, clinical assessment, and radiographic studies. Clinical outcomes before and after the surgery were assessed using Frankel grade and the Japanese Orthopaedic Association (JOA) score. Cervical sagittal alignment was evaluated by C0-2 angle and C2-7 angle based on X-ray.

**Results:**

Nine patients were included in the study. Five patients had schwannoma, three patients had meningioma, and only one patient had neurofibroma. All cases were followed up for 24–42 months (average, 34.2 months). At the last follow-up, three patients improved from Frankel grade C to grade D, two patients from Frankel grade C to grade E, and one patient from Frankel grade D to grade E, while two patients remained stationary at the Frankel grade D. The JOA score of the eight patients were 9.0 (range, 6–17) before surgery and were 14.6 (range, 12–17) at the most recent follow-up (*p* < 0.05). The mean C0-2 angle and the mean C2-7 angle before surgery were 26.2 ± 5.3° and 17.4 ± 13.1°, respectively. At the end of follow-up, the mean C0-2 angle was 25.6 ± 4.8°, and the mean C2-7 angle decreased to 12.7 ± 10.9°. However, this trend did not reach statistical significance (*p* < 0.05). Two patients suffered from cerebrospinal fluid leaks postoperatively. All patients had a satisfactory fusion and did not exhibit a tumor recurrence during the follow-up period.

**Conclusions:**

OCF following gross total resection appears to be a useful surgical procedure for the craniocervical extramedullary tumors requiring C1 facetectomy and does not cause postoperative kyphosis of the upper cervical spine.

## Background

Spinal extramedullary tumors are relatively uncommon, with a reported incidence of 3–10 per 100,000 people and account for two thirds of all primary intraspinal neoplasms [1]. The majority of spinal extramedullary tumors are located in the thoracic region, whereas the occurrence of these lesions is rare in the craniocervical junction (CCJ). In the adult population, the most common spinal extramedullary tumors are benign (WHO grades I and II) and arise from the nerve sheath (approximately 30 %) and from the meninges (approximately 25 %) [2, 3]. Although the clinical presentation is variable depending on its location, radicular pain is the predominant presenting symptom, and motor deficits are present when the lesion is diagnosed later in the clinical course. These patients usually benefit from surgical decompression and resection.

Spinal extramedullary tumors in the CCJ pose considerable difficulties in the operative management regarding surgical approach and technique of fixation. Several surgical approaches have been reported in literature including anterior, lateral, or posterior approaches [4, 5]. As posterior approach facilitates resection and limits postoperative morbidity, it is widely used for surgical resection of spinal extramedullary tumor in CCJ regardless of the tumor location relative to the spinal cord [6]. In some cases, the posterior arch and facet joint of the C1 vertebra need be removed (C1 laminectomy and facetectomy) before extirpation from the craniocervical extramedullary tumor. Unfortunately, a limited laminectomy and facetectomy for resection of spinal cord tumors has been strongly associated with postoperative upper cervical instability or deformity [7–9]. For these reasons, reconstruction of upper cervical stability is considered to be important for preventing postoperative deformity after the resection of spinal extramedullary tumors. Sometimes, however, it is not suitable for placement of C1 screw when C1 pedicle screw trajectory has been destroyed by the tumor or has been broken by C1 facetectomy. Occipito-cervical fusion (OCF) is a demanding and morbid surgical procedure, mainly compromising axial rotation of the head above the trunk but also flexion-extension. This technique can be used in such patients with craniocervical extramedullary tumor requiring removal of the facet joint and posterior elements of C1, which may preclude C1 screw placement [10, 11]. To the best of our knowledge, there are the limited studies regarding surgical management of craniocervical extramedullary tumor and reconstruction of the stability of CCJ. This study was conducted to analyze the clinical manifestation and surgical outcome of patients with craniocervical extramedullary tumor undergoing a gross total resection and OCF only via posterior approach.

## Methods

### Patient cohort

After obtaining an ethical approval from the First Affiliated Hospital of Guangxi Medical University research ethics committee, the authors retrospectively reviewed surgical and clinical databases for the period from January 2008 to July 2011. All of the following inclusion criteria had to be met before the patient was included in this retrospective observational study. The following inclusion criteria were applied: (1) initial surgery for craniocervical extramedullary tumor, (2) gross total resection and occipito-cervical fusion had been performed, (3) minimum 2-year follow-up, and (4) no previous cervical spine surgery. Patients with congenital anomalies in the craniocervical junction were excluded from the study. Written informed consent was obtained from all of the participants involved in the study.

Medical charts were reviewed for data on demographic characteristics, presenting symptoms, and perioperative complications. In most patients, evaluation of neurological function was based on clinical assessment; electromyography (EMG) was also performed in four patients to evaluate nerve root function. Preoperative CT and MRI images were obtained in all patients to evaluate the tumor features and location and the extent of bone destruction. Computed tomography angiography (CTA) was performed in five patients owing to close proximity of the tumors to the vertebral artery (VA). Fusion assessment was based on postoperative plain X-ray films and CT scans. The diagnosis of tumor was confirmed by pathological examination.

The axial MR images were assessed for location of tumor with respect to the spinal cord and were described to a correlate with a clock face. According to previous studies [12], the tumors that were predominantly between “10 and 2 o’clock” were considered “anterior,” those that were either mainly “2 to 4 o’clock” or “8 to 10 o’clock” were considered “lateral,” and those that were from “4 to 8 o’clock” were considered “posterior.”

### Operative technique

All patients in this study underwent occipito-cervical fixation surgery with screw-plate systems and autologous bone grafts for fusion. The plate was fixed to the occiput with bicortical screws and to C2 with polyaxial pedicle screws. Indication for OCF: If C1 laminectomy and facetectomy are performed owing to surgical exposure and C1 is not suitable for screw placement, we recommend OCF in this circumstance. All patients received awake intubation, and the surgical position was prone. The incisions were at midline, from external occipital protuberance to C2 laminar. After adequate exposure of suboccipital and posterior cervical areas, C1 laminectomy and facetectomy were done in all cases depending on the clinical and radiological findings. After bony resection, extradural component of intradural tumor was exposed and removed with standard microsurgical techniques. Before opening the dura mater, the margins of the tumor were delineated with intraoperative ultrasound. To access intradural lesions, the dura was opened by using a longitudinal paramedian incision. Moreover, a T-shaped dural incision was used for dumbbell-shaped tumors. After excision of the tumor, the dura mater was closed in watertight fashion. Occipital condylar screw and C2 pedicle screw fixation was performed after the completion of the intraspinal surgery. Finally, the autologous bone grafts harvested from iliac crest were put between occipital condyle and C2 vertebral. Throughout the procedures, somatosensory evoked potentials and motor evoked potentials were monitored.

### Clinical evaluation

Clinical conditions before and after the surgery were assessed using Frankel grade and the Japanese Orthopaedic Association (JOA) score. The JOA score was assessed before the operation and at the most recent follow-up. The total JOA score assessed motor and sensory functions of four extremities and sphincter, which amounts to a total of seventeen points. The neurologic recovery rate was calculated as follows: (postoperative JOA score − preoperative JOA score)/(full score-preoperative JOA score) × 100. Neurological recovery rate was ranked as excellent (75–100 %), good (51–74 %), fair (25–50 %), poor (0–24 %), or worse (<0 %). Cervical spine lateral radiograph was taken to evaluate cervical sagittal alignment before and after surgery. For the C0-2 angle, an angle between the McRae line and the C2 lower end plate was measured using Cobb method. For the C2-7 angle, an angle between the posterior wall of the C2 vertebral body and the C7 vertebral body was measured using Gore method [13, 14]. Fusion was assessed principally by CT or evaluated by plain radiography of patients who could not take the CT scan during the follow-up period. Satisfactory fusion was defined as successful if two criteria were met: (1) the presence of a homogeneous fusion mass visualized between the graft and bone on CT scans and (2) there was no implant failure or evidence of instability on follow-up image views. Comorbidity and complications were also recorded.

### Statistical analysis

Clinical outcomes were assessed by Frankel grade and JOA scores preoperatively and at the final follow-up. Statistical analysis was performed using the paired *t* test. SPSS for Windows (version 13.0; SPSS, Inc., Chicago, IL, USA) was used for the analysis. A *p* value of less than 0.05 was customarily considered significant.

## Results

There were nine patients (six male and three female) included in the study, with a mean age of 53.1 years (range 37–72 years). The clinical information for these patients is shown in Table 1. Tumor sizes ranged from 2.8 to 6.3 cm (average tumor size 3.4 ± 2.1 cm). Three of these tumors had some form of extradural extension, and six had an extraforaminal extension. Two were located anterior to the spinal cord, while four and three were located posterior and lateral to the spinal cord, respectively. Five patients had schwannoma, three patients had meningioma, and the remaining one patient had neurofibroma. Eight patients had neurological deficits and were graded Frankel C in five cases and D in three. The most common clinical presentation was complaint of neck pain. Six patients presented with neck pain with suboccipital radiation, four patients presented with asymmetrical quadriparesis, and two patients with sensory symptoms like tingling/numbness. The mean preoperative JOA score was 9.0 (range 6–17).

**Table 1:** Summary of clinical data for patients with spinal extramedullary tumors in occipito-cervical junction

Case	Age (years)	Sex	Tumor type	Leson level	Follow-up (months)	Frankel grade		JOA scores			Complications
						Preop.	FFU	Preop.	FFU	NRR	
1	52	F	Schwannoma	Medulla-C1	42	C	E	7	16	90	
2	72	M	Schwannoma	Medulla-C1	24	C	D	7	13	60	
3	49	M	Meningioma	Medulla-C1	41	D	D	10	14	57	
4	56	M	Schwannoma	Medulla-C1	32	D	D	9	14	63	CSF leaks
5	58	M	Schwannoma	Medulla-C1	38	C	D	6	12	55	
6	49	F	Neurofibroma	Medulla-C1	36	E	E	17	17	–	
7	64	F	Meningioma	Medulla-C2	29	C	D	6	13	64	
8	41	M	Schwannoma	Medulla-C2	35	D	E	11	16	83	CSF leaks
9	37	M	Schwannoma	Medulla-C1	31	C	E	8	16	89	

Neurologic recovery rate = (postoperative JOA score − preoperative JOA score)/(full score − preoperative JOA score) × 100

*CSF* cerebrospinal fluid, *FFU* final follow-up, *NRR* neurologic recovery rate, *postop.* postoperative, *preop.* preoperative

**Fig. 1:**
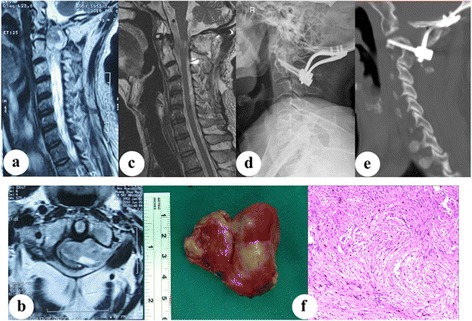
Case 5. This 58-year-old male patient experienced a significant deterioration for his neurological condition several months and presented with neck pain and dysesthesia and was unable to walk properly. **a** preoperative sagittal T2-weighted MR image: spinal extramedullary tumor locates anterior to the spinal cord in the craniocervical region. **b** preoperative axial T2-weighted MR image: this lesion leads to destruction of the C1 posterior arch. **c** postoperative sagittal T2-weighted MR image: the tumor has been removed completely. **d** postoperative X-ray: occiput-C2 fixation has been used by cervical pedicle screws and occipito-cervical plates. **e** CT images at the final follow-up: the success of spinal fusion using autologous iliac bone. **f** postoperative photograph: a well-encapsulated tumor is more than 5 cm across, and pathology report confirms the diagnosis of schwannoma

In the current series, gross total resection was attempted and achieved in every patient. All patients were available for follow-up with an average follow-up of 34.2 months (24–42 months). According to the Frankel classification, no patient had neurological deterioration postoperatively. At the last follow-up, six patients had improved (three patients improved from Frankel grade C to grade D, two patients from Frankel grade C to grade E, and one patient from Frankel grade D to grade E), while two patients remained stationary at the Frankel grade D. There was a statistical significance was found between the Frankel grade before surgery and at the most recent follow-up (*p* < 0.05). For the JOA scores, one neurologically intact patient remained same after surgery, and the remaining patients had improvement of the JOA scores at the last follow-up (9.0 ± 3.5 vs. 14.6 ± 1.7, *p* < 0.05). According to neurological recovery rate, three patients were excellent and five patients were good. The mean C0-2 angle and the mean C2-7 angle before surgery were 26.2 ± 5.3° and 17.4 ± 13.1°, respectively. At the end of follow-up, the mean C0-2 angle was 25.6 ± 4.8°, and the mean C2-7 angle decreased to 12.7 ± 10.9°. However, this trend did not reach statistical significance (*p* < 0.05). In this case series, there were no cervical kyphotic deformity and serious complications during the follow-up. Two patients suffered from cerebrospinal fluid (CSF) leaks because of a dural breach, which were successfully treated by conservative management of CSF leaks within 7 days including neck wrapping and strict bed rest. No surgical revision was required related to the complications. All patients had a satisfactory fusion and did not exhibit a tumor recurrence during the follow-up period (Fig. 1a-f).

## Discussion

Treatment of craniocervical extramedullary tumor is challenging as CCJ presents a unique, complex, biomechanical interface between the cranium and the upper cervical spine [15]. Various surgical approaches have been advocated for the extramedullary tumor in CCJ, depending on the anatomical location of the tumor relative to the spinal cord [12]. Tumors situated posterior or posterolateral to the spinal cord or the brainstem can be safely resected via a posterior midline suboccipital approach combined with C1 laminectomy. The optimal surgical approach to the anterior and anterolaterally located tumors still remains debatable [4, 16]. Theoretically, the transoral route is considered as the best approach for ventral tumors, which provided a direct and natural approach to the CCJ [17]. However, this approach has several drawbacks, including CSF leak, subsequent infections, velopharyngeal insufficiency, and limited lateral access [18]. The lateral approaches to extramedullary tumor in CCJ include the anterolateral or the extreme lateral approach and the posterolateral or the far lateral approach. Several authors reported that these approaches provided an adequate exposure for tumor resection in comparison with a conventional anterior approach [19, 20]. However, the lateral approaches might be technically challenging as risk of accessory nerve or vertebral artery injury and venous plexus hemorrhage. Recently, Fong [21] and Bydon [22] reported that patients with tumor could be successfully treated with endoscopic endonasal and others minimally invasive approaches, which offer the potential benefits of less blood loss and quicker recovery, and it prevents postoperative instability from extensive bone resection while maintaining spinal structural integrity. However, the major shortcomings of minimally invasive approaches are the limited space for manipulation and the restricted view [23]. Additionally, minimally invasive technique needs to evolve over time, and the learning curve may be quite steep. Noticeably, Ahn and colleagues in a retrospective study of patients that underwent surgical resection of spinal extramedullary tumors via a posterior approach reported no difficulties in removing ventrally located extramedullary tumors [24]. Actually, complete excision in ventral spinal extramedullary tumors is also not easy via an anterior approach because it seems difficult to use this approach for a tumor outside the intervertebral foramen in the upper cervical cord. To overcome these difficulties, the lateral facet joint must be excised by posterior approach, and an adequate visual field of the ventrally located tumors must be maintained when approaching them. Thus, a single posterior approach was used in our patients by this method, regardless of the location of the tumor relative to the spinal cord. Consistent with the previous findings, we found it was safe and effective for resecting ventrally located tumors through a purely posterior approach.

Regardless of the surgical approach, complete resection of the spinal extramedullary tumors is one of the primary surgical goals. Numerous studies have shown a positive association between gross total resection and favorable outcomes [12, 25]. Gottfried and colleagues [2] achieved complete resection in 92 % of their cases series and found that patients who underwent gross total resection were more likely to remain disease free than patients who underwent partial or subtotal resection. Consistent with Gottfried’s study, Slin’ko [16] noted a lower recurrence rate in patients who underwent gross total resection. In the current series, five patients had schwannoma, three patients had meningioma, and one patient had neurofibroma. Although the tumors size larger than 3 cm and ventral location were technically difficult [5], the gross total resection was achieved in every patient in our study. Several factors may be responsible for this condition. First, the vertebral artery involvement was all widely accepted as an important factor against radical resection. Fortunately, our patients had no artery involvement which may significantly affected radical resection. Second, the length and extent of laminectomy or the removal of facet joint of C1 are directly associated with the safety and effectiveness of complete resection. The concomitant fusion procedures may permit more radical resections without concern for iatrogenically induced spinal instability. Postoperative clinical and radiographic improvements were maintained during the follow-up period. The rate of neurological recovery was 70.1 %, which is in accordance with the findings of previous reports [26]. At the final follow-up, no evidence of recurrence was observed in our patients. The result of the present study confirmed the previous findings that gross total resection was the most important treatment variable influencing long-term outcomes and recurrence rates in patients with spinal extramedullary tumors [24, 27].

In order to provide a surgical window and expose the extramedullary tumor at the CCJ, it is necessary to perform a limited craniectomy of the inferior aspect of the occiput and resection of posterior elements and facet joint of C1. Concomitant spinal fusion and stabilization may be required depending on the amount of bony resection. Sciubba [7] and Mcgirt [8] reported that patients who undergo laminectomy at high stress regions like the CCJ will probably have an increased risk for postoperative CCJ instability as biomechanical mechanism. In cadaveric and clinical study, some authors found the cervical instability after resection of more than 30–50 % of the facet joint [28, 29]. Furthermore, spinal cord lesions alone, without surgical destabilization, can lead to spinal instabilities and deformities in both children and adults as involvement of the anterior horn cells causes muscle denervation and weakness [30]. For these reasons, spinal stability should be evaluated in cases of spinal tumor extirpation from the CCJ requiring C1 facetectomy. Fusion procedures are recommended to perform if instability is evident. Nevertheless, it is cautious to determine whether OCF is necessary in these patients with craniocervical extramedullary tumor as it may sacrifice the motion of the occipital and C1-2 complex. The ndication of OCF have documented by several surgeons, however, which is still controversial [31, 32]. If the integrity of occipital-C1 facet capsule is possible, we usually perform C1-2 fusion so that occipital-C1 motion is preserved. In other words, we recommend OCF for instability if removal of the C1 facet joint, either unilateral or bilateral, and C1 is not suitable for placement of screws. In our study, the mean C0-2 angle and the mean C2-7 angle before surgery were 26.2 ± 5.3° and 17.4 ± 13.1°, respectively. At the end of follow-up, the mean C0-2 angle was 25.6 ± 4.8°, and the mean C2-7 angle decreased to 12.7 ± 10.9°. No evidence of instability at the lower adjacent motion segment and kyphosis of the upper cervical spine were observed until a mean follow-up of 34.2 months. The results demonstrate that OCF is valuable for use in patients who require the extramedullary tumor resection and occipito-cervical stabilization by one-stage posterior approach.

There are several limitations in our study. First, sample size is small, which are related to the extremely rare and unusual patients with such conditions. Second, the mean follow-up period (34.2 months) was relatively short, and various pathological diagnoses were included. Our results will need to be confirmed in larger series with longer follow-up.

## Conclusions

Guidelines for OCF stabilization after resection of extramedullary tumor at the CCJ have been still unclear, and the literature includes very few reports of clinical experience in this area. On the basis of our findings in the clinical series, we recommend that OCF be performed in cases of tumor extirpation from the CCJ requiring C1 facetectomy, which may prevent the postoperative kyphosis of the upper cervical spine.

## Consent

Written informed consent was obtained from all patients enrolled in the investigation. The study protocol conformed to the ethical guidelines of the 1975 Declaration of Helsinki and the guidelines of the regional ethical committees of Zurich, Switzerland, and Basel, Switzerland.

## Abbreviations

CCJ: craniocervical junction
CSF: cerebrospinal fluid
JOA: Japanese Orthopaedic Association
OCF: occipito-cervical fusion
